# Betanodavirus-like particles enter host cells via clathrin-mediated endocytosis in a cholesterol-, pH- and cytoskeleton-dependent manner

**DOI:** 10.1186/s13567-017-0412-y

**Published:** 2017-02-08

**Authors:** Runqing Huang, Guohua Zhu, Jing Zhang, Yuxiong Lai, Yu Xu, Jianguo He, Junfeng Xie

**Affiliations:** 1 State Key Laboratory of Biocontrol, Institute of Aquatic Economic Animals and Guangdong Province Key Laboratory for Aquatic Economic Animals, School of Life Sciences, Sun Yat-sen University, Guangzhou, 510006 China; 2 Department of Nephrology, Guangdong Academy of Medical Sciences, Guangdong General Hospital, Guangzhou, China; 3 South China Sea Fisheries Research Institute, Chinese Academy of Fishery Sciences, Guangzhou, China; 4 School of Marine Sciences, Sun Yat-sen University, Guangzhou, China

## Abstract

Betanodavirus, also referred to nervous necrosis virus (NNV), is the causative agent of the fatal disease, viral nervous necrosis and has brought significant economic losses in marine and freshwater cultured fish, especially larvae and juveniles. Here, we used an established invasion model with virus-like particle (VLP)-cells, mimicking orange-spotted grouper nervous necrosis virus (OGNNV), to investigate the crucial events of virus entry. VLP were observed in the perinuclear regions of Asian sea bass (SB) cells within 1.5 h after attachment. VLP uptake was strongly inhibited when cells were pretreated with biochemical inhibitors (chlorpromazine and dynasore) blocking clathrin-mediated endocytosis (CME) or transfected with siRNA against clathrin heavy and light chains. Inhibitors against key regulators of caveolae/raft-dependent endocytosis and macropinocytosis had no effect on VLP uptake. In contrast, disruption of cellular cholesterol by methyl-β-cyclodextrin or reduction of cholesterol fluidity by Cholera toxin B subunit significantly decreased VLP entry. Furthermore, VLP entry is dependent on low pH and cytoskeleton, demonstrated by inhibitor (chloroquine, ammonia chloride, cytochalasin D, wiskostatin, and nocodazole) perturbation. Therefore, OGNNV VLP enter SB cells via CME depending on dynamin-2, cholesterol and its fluidity, low pH, and cytoskeleton. In addition, ten more cell lines were screened for VLP entry and VLP can only enter NNV-sensitive cells, GB and SSN-1, via CME, indicating that CME is the common endocytosis pathway for VLP. These results may provide the data for NNV entry without the influence of the viral genome, an ideal model for exploring the behaviour of betanodavirus in cells, and valuable references to vaccine development.

**Electronic supplementary material:**

The online version of this article (doi:10.1186/s13567-017-0412-y) contains supplementary material, which is available to authorized users.

## Introduction

To successfully infect host cells, viruses must first bind to cell surface proteins, carbohydrates, or lipids. Interactions of viral structural proteins with cellular receptors are often specific and multivalent. These interactions activate the cellular signaling pathways that respond by internalizing the viruses using one of several endocytic mechanisms, including clathrin-mediated endocytosis (CME), caveolae/raft-dependent, non-clathrin-caveolae/raft-dependent pathways, macropinocytosis, and a variety of other still poorly characterized mechanisms [1].

Clathrin-mediated endocytosis is the best characterized pathway of virus invasion and a classical endocytic mechanism, which most viruses use as the primary route of internalization [2]. A large number of viruses that enter host cells through CME have been identified, such as influenza virus [3], African swine fever virus [4], dengue virus serotype 2 [5], Singapore grouper iridovirus (SGIV) [2] and so on. During this endocytic process, clathrin is assembled on the plasma membrane to form a clathrin-coated pit (CCP). CCP then invaginates to form a clathrin-coated vesicle (CCV) containing the internalized viruses. During CCV budding, the membrane to be internalized and the size of the future vesicle are selected before invagination during cargo recruitment, suggesting that bending of a dynamic preassembled clathrin coat is involved in this process [6]. The vesicle subsequently sheds its clathrin coat and transports into acidic endosomal and lysosomal compartments and the *trans*-Golgi network [7]. A series of essential molecules, including Eps15, dynamin, and adapter protein AP2, are involved in this pathway. Chlorpromazine (CPZ), a biochemical inhibitor, can inhibit the clathrin-mediated uptake of viruses by preventing the assembly of CCP at the plasma membrane [8]. Low pH in the endosomes and lysosomes triggers viral penetration. Thus, the CME pathway is sensitive to pH changes in these low-pH compartments and biochemical reagents, such as chloroquine and ammonium chloride (NH_4_Cl), can inhibit the viral infection [9].

Another well-characterized pathway is caveolae/raft-dependent endocytosis, an alternative to CME. In this mechanism, viruses bind to a specialized membrane domain composed of caveolin and associated with high levels of cholesterol and sphingolipids [10], called caveolae. Normally, caveolae on most cell surfaces exhibit little motility and dynamics. However, a signal cascade caused by the activation of tyrosine kinases can result in slow but efficient internalization [11]. Many reports show that endocytosis mediated by caveolae requires unique structural and signaling machinery [12]. Ligands internalized via caveolae will later be delivered to several different compartments. For example, cholera toxin (CTx) are delivered to caveosomes, the caveolin-1-positive, pH-neutral endocytosis compartments, and then transported to the Golgi complex and the endoplasmic reticulum [13]. The use of caveolae/raft-dependent endocytosis for the entry of other viruses into host cells has been demonstrated, such as simian virus 40 (SV40) [14], echovirus 1 (EV1) [15], foot-and-mouth disease virus [16], infectious spleen and kidney necrosis virus (ISKNV) [17], among others.

Lipid rafts are specialized membrane microdomains that are enriched in sphingolipids, cholesterol, Src family protein kinases, and glycosylphosphatidylinositol-anchored proteins; thus, cholesterol is essential in lipid raft membranes [18]. For viruses, cholesterol-enriched membrane microdomains play an important role in multiple stages of virus life cycle that includes entry, fusion, replication, assembly and budding [19]. Because caveolae are associated with cholesterol-rich lipid rafts, disruption of membrane cholesterol severely inhibits virus entry via caveolae/raft-dependent endocytosis. However, accumulating evidence shows that cholesterol depletion also inhibits CME [1] whether in studies on virus entry or toxin and drug metabolism. For example, transactivator of transcription (TAT)-modified and unmodified lipoplexes are mainly internalized via a cholesterol-dependent CME [20]. As for virus entry, a cholesterol-dependent CME is also identified in many studies, such as Japanese encephalitis virus (JEV) [21], adenovirus type 2 [22], chikungunya virus [23], Rhesus rhadinovirus [24], among others. Besides the presence of cholesterol, the fluidity of cholesterol or in other words the fluidity of the membrane is also important to virus entry. An increase in the membrane fluidity of lipid rafts was observed in the early stages of JEV infection [21]. Therefore, an increase in membrane fluidity will theoretically facilitate virus entry into the host cells, which was observed in human immunodeficiency virus type 1 infection [25] and hepatitis C virus (HCV) infection [26]. However, conflicting results have also been observed. Phenothiazines are drugs that inhibit HCV entry by increasing the fluidity of cholesterol-rich membranes [27]. Nevertheless, virus entry and internalization are sensitive to the plasma membrane fluidity. To decrease the membrane fluidity, CTx is normally used to bind sphingolipid GM1, reducing the diffusion of other lipid molecules in the same membrane [28].

Virus-like particles (VLP) are mono- or multi-protein structures that mimic the organization and conformation of authentic native viruses but lack the viral genome, potentially yielding safer vaccine candidates than inactive viruses [29]. Attenuation or inactivation is not required for VLP vaccine and this step is particularly important since epitopes are commonly modified by inactivation treatments [30]. Compared with individual proteins or peptides, VLP present conformational epitopes similar to the native virus [31]. Owing to the similarity of surface protein structure to the native viruses, corresponding VLP are used as substitutes for studies of highly virulent viruses [32] or as a model for antiviral drug screening [33]. Since the entry process is based on the interaction of viral structural proteins and cellular components without the participation of the viral genome, it is reasonable to use VLP to study the virus entry process with the advantage of easy manipulation [34]. The  indirect immunofluorescence assay (IFA) can be used to visualize viral structural proteins during virus entry and trafficking in the target cells. In contrast to infectivity or reporter detection, the assay using IFA to detect VLP location after VLP entry does not rely on the expression of any viral and reporter genes, but instead directly visualizes the accumulation of viral particles in cells as an indicator of successful viral entry and trafficking in cells [35].

The Nodaviridae family contains two genera: *Betanodavirus*, the viruses in which predominantly infect fish, and *Alphanodavirus*, the viruses in which mostly infect insects [36]. Betanodaviruses are the causative agents for disease of viral nervous necrosis (VNN), an infectious neuropathological condition characterized by necrosis of the central nervous system, including the brain and retina, which shows clinical signs that include abnormal swimming behaviour and darkening of the fish [37]. VNN is capable of causing massive mortality in the larvae and juvenile populations of more than 40 marine and freshwater teleost species [38], suggesting their strong infectivity to a wide range of hosts. Compared with the intensive studies on the alphanodavirus [39], viruses in another genus in the same family, research on the entry process of betanodavirus are still a rarity. Current studies have only showed that the cellular heat shock cognate protein 70 (HSP70) [40] and cell surface sialic acid [41] are essential for NNV infection, endosomal acidification is required for effective infection [42], and NNV entry utilizes both micro- and macropinocytosis pathways [41]. Therefore, investigating the mechanism of NNV entry into host cells is essential and critical to understanding the true behaviour of betanodavirus infection. The capsid protein of orange-spotted grouper nervous necrosis virus (OGNNV) and dragon grouper nervous necrosis virus (DGNNV) expressed in *Escherichia coli* also forms VLP that morphologically resembles native virus [43, 44]. The VLP can block the attachment of the native virus to the surface of striped snakehead (SSN-1) cells, thus restricting virus infection [45] or even enter sea bass (SB) cells at the same dynamics as that of native virus [46]. This phenomenon suggests that the outer shell of VLP is structurally indistinguishable from native virus and recognized by the putative cellular receptor(s).

In the present study, we used VLP originated from OGNNV (RBS) and SB cells as a model for virus entry to examine the pathway and important factors of betanodavirus entry into host cells using perturbation, such as biochemical inhibition or siRNA silencing, and IFA to detect the route of VLP entry. C-terminal green fluorescent protein-tagged VLP (CGV) was also used to locate VLP in cells in real-time and study membrane fluidity. In addition, we screened other types of cells for VLP entry ability. Our results not only contribute greatly to understanding betanodavirus entry and pathogenesis but also provide new insights into vaccine design.

## Materials and methods

### Cell culture

The SB fibroblast cell line derived from *Lates calcarifer* larvae was obtained from Temasek Life Sciences Laboratory of the National University of Singapore [44]. SB cells are sensitive to OGNNV and were used for the cell entry assay. The cells were grown in minimal essential medium (MEM, Gibco, USA) supplemented with 10% fetal bovine serum (FBS, Gibco). SSN-1 derived from *Channa striatus* larvae and GB derived from the brain of *Epinephelus coioides* were grown in L15 and DMEM (Gibco) respectively. Mandarin fish fry-1 (MFF-1) cells derived from *Siniperca chuatsi* fry [47], fathead minnow (FHM) cells derived from *Pimephales promelas*, and *epithelioma papulosum cyprini* (EPC) cells derived from *Cyprinus carpio* were grown in DMEM and M199 respectively. All fish cell lines were grown at 26 °C supplemented with 10% FBS. SF9 derived from *Spodoptera frugiperda* and Drosophila Schneider 2 (S2) derived from *Drosophila melanogaster* were grown at 28 °C in Grace’s Insect Medium and Schneider’s Insect Medium (Gibco) supplemented with 10% FBS respectively. Hela, 293T and baby hamster kidney (BHK) cells were grown at 37 °C in DMEM supplemented with 10% FBS. For IFA or ELISA following biochemical inhibitor analysis, the cells were seeded on coverslips in 12-well plates or cultured in 96-well plates for 14 or 18 h to achieve 70% confluence.

### Antibodies, reagents, and biochemical inhibitors

A polyclonal antibody, mouse anti-VLP sera (total immunoglobulin), were produced from our laboratory. Alexa Fluor 488 donkey anti-mouse IgG, Alexa Fluor 594 donkey anti-mouse IgG, and Alexa Fluor 488 donkey anti-goat IgG was from Molecular Probes (Invitrogen, USA). DAPI, Goat anti-biotin antibody, chlorpromazine (CPZ), dynasore, methyl-β-cyclodextrin (MβCD), cholesterol, genistein, wortmannin, Cholera toxin B subunit (CTB), ML-7, NSC23766, rottlerin, IPA-3, chloroquine, ammonia chloride (NH_4_Cl), cytochalasin D (CytD), wiskostatin, and nocodazole were purchased from Sigma-Aldrich (Merck, USA). All drugs were dissolved, prepared, used in certain concentrations and stored at the indicated temperature as shown in Table 1.

**Table 1:** **The preparation details of all drugs used in this study**

Drug	Solventt	ST (°C)^a^	WC(μM)^b^	MC(μM)^c^
Chlorpromazine	ddH2O	4	1, 7, 70	700
Dynasor	DMSO	4	1, 10, 80	800
M-β-CD	ddH2O	4	500, 1000, 2000	2000
Genistein	DMSO	−20	10, 50, 100	100
Wortannin	DMSO	−20	1, 10, 50	50
ML-7	DMSO	−20	1, 20, 40	40
NSC23766	DMSO	−20	1, 20, 40	40
Rottlerin	DMSO	4	1, 20, 40	40
IPA-3	DMSO	−20	1, 20, 40	40
Chloroquine	ddH2O	4	10, 100, 1000	1000
NH_4_Cl	ddH2O	4	1000, 5000, 50 000	50 000
Cytochalasin D	DMSO	−20	1, 10, 77.8	77.8
Wiskostatin	DMSO	−20	1, 10, 50	50
Nocodazole	DMSO	−20	1, 10, 50	160

All drugs used in this study are listed.

^a^ ST means storage temperature.

^b^ WC indicates the working concentrations used in this study.

^c^ MC indicates the max concentration that can be used without cytotoxicity.

### Cytotoxicity assay

To optimize the working concentration of the inhibitors, the cytotoxicity of the drugs to SB cells were evaluated. SB cells grown in 96-well plates were treated with the inhibitors at different concentrations for 4 h. Then cell viability was assayed using CellTiter 96^®^ AQueous One Solution Cell Proliferation Assay (Promega, USA) according to the manufacturer’s instructions. The concentration of all drugs employed in this study did not cause significant adverse effects on cell viability (Table 1).

### VLP production and cell entry assays

OGNNV VLP, including the native form of VLP (RBS) and the modified VLP (CGV), were produced as described [44]. In brief, a volume of 80 mL seed culture grown overnight at 37 °C was inoculated equally into 8 flasks of 1 L LB-broth medium supplemented with 100 μg/mL ampicillin. When the cell density reached an OD600 of 0.3–0.4, the culture was cooled down to 30 °C for RBS or 23 °C for CGV and 0.9 mM IPTG was added for induction. After induction of 2.5 h at 30 °C for RBS or 8 h at 23 °C for CGV, cells were harvested by centrifugation and resuspended in 100 mL lysis buffer, followed by sonication and centrifuged at 40 000 × * g* for 20 min. The supernatant was ultracentrifuged at 250 000 × *g* for 1 h against a 30% sucrose cushion. The pellet was resuspended in 4 mL of PBS (pH 8.0) and further purified by ultracentrifugation against a 10–40% (w/w) sucrose gradient at 250 000 × *g* for 3 h. The fractions containing fine structured and highly pure VLP were diluted with PBS and ultracentrifuged at 250 000 × * g* for 1 h to remove the sucrose. The pellets were resuspended with PBS and the VLP (RBS or CGV) were verified by Transmission Electron Microscopy (JOEL JEM-1400, Japan). Purified VLP were applied to SDS-PAGE and BCA Protein Assay Kit (Thermo, USA) to determine the concentration and stored at −80 °C until use.

Virus-like particles entry (invasion) assays were performed using SB or other indicated cells as described [46]. In brief, the cells were seeded on coverslips in 12-well plates or cultured in 96-well plates for 18 or 24 h to achieve 70% confluence, pretreated with the indicated inhibitors at the indicated temperature (26 °C for fish cells, 28 °C for insect cells, and 37 °C for mammalian cells) and concentration (Table 1) for 1 h, and then incubated with RBS or CGV in the presence of the inhibitors for 1 h on ice. After that, the cells were washed three times with pre-cooling PBS, shifted to 28 °C to start entry, and visualized directly by fluorescent microscopy for CGV entry kinetics, or fixed for IFA followed by fluorescent microscopy, or collected for ELISA at the indicated times. In the untreated (positive) or mock-infected (negative) controls, the cells were subjected to no inhibitor treatment or no VLP incubation respectively. The entry assay of each inhibitor was repeated at least three times.

### Gene cloning, siRNA generation and cell transfection

According to the clathrin sequences available from fish (accession num: HQ441139, XM_010735171, XM_010735171, XM_014407499), degenerated primers for clathrin heavy chain (CHC) and clathrin light chain (CLC) were respectively designed and synthesized (Table 2). Total RNA was extracted from SB cells using RNeasy Mini Kit (Qiagen, Germany) and reverse-transcribed into cDNA using the First Strand cDNA Synthesis Kit (Toyobo, Japan) according to the manufacturer’s instructions. cDNA from SB cells were used as the template for PCR to amplify the conserved sequence of clathrin and the PCR products were sequenced to confirm the partial CDS. Based on the CDS, RACE primers were designed (Table 2) and the full length sequences of sbCH and sbCL were obtained by SMARTer RACE 5′/3′ kit (Takara, Japan) according to the manufacturer’s instructions.

**Table 2:** **Primers and siRNA used in this study**

Name	5′ → 3′
*Degenerated primers*	
CH-F1	ATCTCWGCHGAYAGYGCCATCAT
CH-F2	ATCAGYGSDGAGACCATHTTTGT
CH-F3	GCYAAGATCTACATYGACAGCA
CH-F4	AGGAAGCHAARCTIACHGACCA
CH-R1	CTCTGYYTCCAGCGGTTGTT
CH-R2	GAGITAYTCYCTCATGACITGYATR
CH-R3	CAGGCAGGCBGCAAARCACT
CL-F1	GATTTTGACATGCTGAACGC
CL-F2	ATTGAAAACGACGAAGGCTT
CL-F3	AATGGAGAGCTTCATGGGGA
CL-R1	TTCAGGGAGATGAGGACCGA
CL-R2	CCTGCTTGCTGGACTTGGG
*Race primers*	
CHR5-R1	CTGGGTTTCTGACAGTGCT
CHR5-R2	CCTGGTCACACTGTCCTCTCTC
CHR3-F1	TCAACTTCTTCAGCAAGGTG
CHR3-F2	ACTATCAGGCACTGAGGACCTC
CLR5-R1	TTCTTGGCCCAGCAAGAGAGC
CLR5-R2	CCGCTGGAAACGGTGTCGG
CLR3-F1	AACCCCAAGTCCAGCAAGCAG
CLR3-F2	CATGCGCTCCGTCCTCATCT
*siRNA sequence*	
CHC1-S	GUUAAGGAGGCCAUUGACUCC
CHC1-A	AGUCAAUGGCCUCCUUAACCA
CHC2-S	GUACAGAACCACAACAACAAA
CHC2-A	UGUUGUUGUGGUUCUGUACUG
CHC3-S	GCAUUUAAUUAUUGUAUAUUC
CHC3-A	AUAUACAAUAAUUAAAUGCAU
CLC1-S	GGUACUUUCUAUAACUACUAC
CLC1-A	AGUAGUUAUAGAAAGUACCUG
CLC2-S	GCUUAUGCAGCAAUUUCCAAU
CLC2-A	UGGAAAUUGCUGCAUAAGCAU
CLC3-S	GUUUGUAUGUGUGUUUAAACC
CLC3-A	UUUAAACACACAUACAAACGG
IR1-S	AAUAGUCAUUGUGUAUGGCCA
IR1-A	GCCAUACACAAUGACUAUUUG
IR2-S	AAUGGCUAAGGUUUUGAUCAC
IR2-A	GAUCAAAACCUUAGCCAUUAU

siRNA against sbCH (siCH) and sbCL (siCL) were designed based on the full-length mRNA sequences (accession Num: KX989459, KX989460) using the online tools siRNA Wizard v3.1 [48] and siDirect V2.0 [49]. siRNA with an irrelevant sequence to all known genes (siIR) were also designed and the sequences of the sense and antisense siRNA strands were displayed in Table 2. T7 RiboMAX™ Express RNAi System (promega) was used to synthesize siRNA by in vitro transcription according to the manufacturer’s instructions. siRNA against the same target were used as an equally mixed pool in transfection experiments. Pools containing 300 nM siRNA of sbCH, sbCL or siIR were transfected into SB cells using Lipofectamine^®^ 2000 Transfection Reagent (Invitrogen) according to the manufacturer’s instructions, respectively. The RNA level of *clathrin* was tested by quantitative RT-PCR to evaluate the silence efficiency before the VLP entry assays were performed.

### Immunofluorescence assay

Cells were washed twice with PBS, fixed with 4% paraformaldehyde for 15 min, permeated with 0.2% Triton X-100 for 10 min, and then blocked with 5% BSA for 1 h. Cells were incubated at 4 °C overnight with primary antibodies such as mouse anti-VLP sera (polyclonal antibody produced in-house, total immunoglobulin) or goat anti-biotin monoclonal antibody at 2000-fold dilutions. Subsequently cells were washed three times with PBS for 15 min and incubated for 1 h at room temperature with secondary antibodies such as Alexa Fluor 488 donkey anti mouse IgG or Alexa Fluor 594 donkey anti goat IgG at 4000-fold dilutions. Cell nuclei were labeled with DAPI (1:1000, Sigma) diluted in PBS for 10 min and then cells were washed three times with PBS. For identification of entry and trafficking of RBS in cells, images were acquired using a laser scanning confocal fluorescence microscope (TCS-SP5, Leica Microsystems Wetzlar GmbH Inc., Germany). Images were acquired randomly from 5 different fields of view per coverslip to get more than 20 cells and to allow observing the signals of targets and nuclei fluorescence by Leica Application Suite (2.6.0 build 7266). For real-time tracking of CGV, a fluorescent inverted microscope (Nikon Eclipse Ti-U, Japan) was used to continuously acquire images utilizing Nikon NIS Elements imaging software (Br2 v3.21). All experiments were performed in triplicate. The results are expressed as the mean ± standard deviation (SD).

### ELISA

Six wells of cells in a 96-well plate treated with the same condition in entry assays were washed thoroughly with PBS, lysed with 20 μL of lysis buffer in each well and combined as one sample. Endogenous β-actin was used as an internal normalization control for ELISA. Three-hundred microliters of 30-fold diluted lysate for VLP detection or 100-fold diluted lysate for β-actin detection were coated equally in three wells of a 96-well microtiter plate at 4 °C overnight, respectively. The plate was conducted to room temperature and washed three times with 200 μL PBS. After blocking with 5% bovine serum albumin (BSA) in PBS for 1 h at room temperature and washing three times (all washes below indicate triplicate washings) with 200 μL PBS containing 0.05% Tween20 (PBST), the plate was incubated with in-house produced mouse anti-VLP antiserum at a dilution of 1:2000 in PBS for 1 h. The plate was washed and peroxidase-conjugated goat anti-mouse IgG (Sigma) at a dilution of 1:3000 in PBS was added and incubated for 1 h. Following thorough washings (five times), 100 μL of OPD substrate (Sigma) was added and the color development was conducted at room temperature. The reaction was stopped by the addition of 4 M sulfuric acid and the absorbance at 492 nm was determined. The OD_492_ of each sample including treated and untreated cells was normalized with the corresponding OD_492_ of β-actin to obtain the relative VLP quantity. The entry efficiency was generated as the percentage of treated cells with incorporated VLP relative to that for untreated cells. The VLP entry efficiency of untreated cells was arbitrarily set as 100%. The data shown are the mean ± SD of the results from three independent experiments. Statistical differences of VLP content from different groups were assessed by paired Student’s *t* tests. Numerical results are presented as mean ± SD with 95% confidence intervals and *p* < 0.05 was considered statistically significant (*, *p* < 0.05; **, *p* < 0.01).

## Results

### The kinetics of VLP entry into SB cells

**Figure 1:**
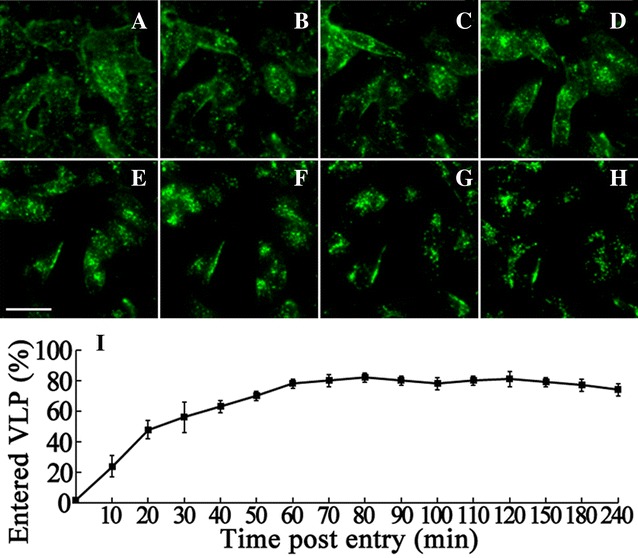
**The entry process and the entry kinetics of CGV in SB cells.** The entry process of CGV. CGV entry was performed on SB cells as mentioned above and at indicated times, 0 (**A**), 10 (**B**), 20 (**C**), 30 (**D**), 60 (**E**), 90 (**F**), 120 (**G**), and 240 min (**H**) after entry start, live cells were visualized by fluorescent microscopy to locate the CGV position. The bar in **E** indicates 25 μm. **I** The entry kinetics of CGV. CGV entry was performed as mentioned above and at the indicated times, cells were lysated for ELISA to evaluate the CGV quantity.

It has been demonstrated that RBS and CGV can enter SB cells rapidly as the native virus [46]. Moreover, DGNNV VLP can block the cell attachment and infection of native virus [45]. We speculated that VLP use the same receptor(s) to enter cells as the native betanodavirus, suggesting that VLP and native viruses enter cells through the same pathway. To study the dynamics of VLP entry, CGV was used to track the locations of VLP in SB cells by fluorescent microscopy during different entry times. After incubation with CGV at 4 °C for 30 min, the cells were plated rapidly at 28 °C to initiate entry. The cells were observed at the indicated time points (0, 10, 20, 30, 60, 90, 120, and 240 min) without fixation. As shown in Figure 1, CGV entry into SB cells is a quick process similar to the wild-type OGNNV [44]. CGV bound to the cell plasma membrane without endocytosis at 4 °C (Figure 1A, 0 min). Entry was rapidly initiated when the temperature was increased to 28 °C (Figure 1B, 10 min), followed by a time-dependent increase of fluorescent signal in the cytoplasm with increasing entry times (Figures 1B–G, 10–120 min). CGV gathered around the nucleus after 1 h post-invasion (hpi) (Figure 1E). The fluorescent intensity of CGV remained similar between 1.5 (Figure 1F) and 4 hpi (Figure 1H), suggesting that CGV entry was complete within 120 min after attachment. The entry process of CGV was from cell plasma membrane to perinuclear spot-shape areas. Interestingly, CGV was likely to diffuse from perinuclear areas after 4 hpi when compared with the CGV location at 120 min (Figure 1G).

The quantities of entered VLP at different time points were also evaluated by ELISA and the entry kinetics of CGV was determined. The percentage of CGV entry of SB cells increased rapidly within 10 min (24%), reaching the half-maximal level (50%) at 30 min, and plateauing after 90 min (Figure 1I). According to the fluorescent and ELISA results of CGV entry, the entry process is quick.

### Clathrin-mediated endocytosis is involved in RBS entry

To identify the endocytosis pathway involved in RBS entry, cell perturbation assays of biochemical inhibition were performed to evaluate the effect on VLP entry. All biochemicals used were tested on SB cells by cytotoxicity assays to obtain the maximum working concentration (Table 1).

**Figure 2:**
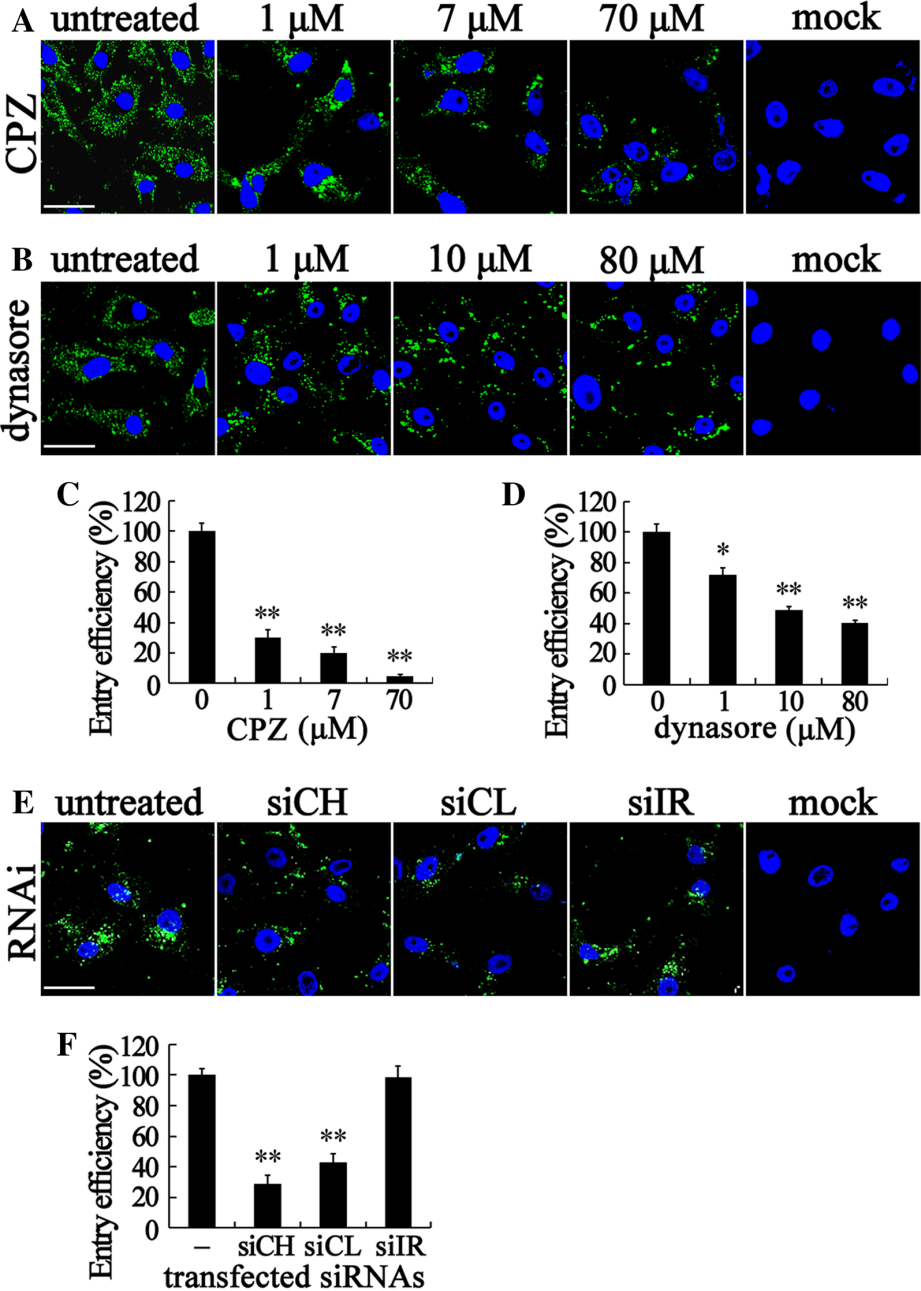
**RBS entry depends on CME and dynamin. A**–**D** CPZ and dynasore reduce the entry and trafficking of RBS to the perinuclear region of SB cells. SB cells were pretreated with CPZ or dynasore at the indicated doses for 1 h, or left untreated, then incubated with RBS for 2 h, and fixed for IFA (**A**,** B**) or collected for ELISA (**C**, **D**). In the untreated or mock-infected controls, cells were subjected to no inhibitor treatment or no RBS incubation respectively. RBS (green) in cells were visualized by IFA and cell nuclei (blue) were stained by DAPI. The entry efficiency was examined by ELISA and the data shown are the mean ± SD of the results from three independent experiments. **E**, **F** Inhibition of sbCH and sbCL by RNAi reduced the entry of RBS. siRNA specific to sbCH (siCH), sbCL (siCL) and siRNA with irrelevant sequence (siIR) were transfected to SB cells 48 h prior to VLP entry assay. After the entry assay, cells were fixed for IFA (**E**) or collected for ELISA (**F**). The bars in (**A**, **B**, **E**) indicate 25 μm. *, *p* < 0.05, **, *p* < 0.01.

During CME, CCP assemble on the cytoplasmic side of the plasma membrane in response to internalization signals from the receptor. The cationic amphiphilic agent CPZ inhibits CME by causing misassembly of CCP [50]. Therefore, CPZ was used to analyze the role of the CME pathway during RBS entry into SB by adding different concentrations of CPZ. The RBS in cells were determined by IFA. As shown in Figure 2A, CPZ significantly reduced the fluorescent signal of RBS compared with no treatment cells from 1 to 70 μM. In addition, CPZ reduced the absolute number of viral particles reaching the perinuclear area. Through ELISA quantitation, we found that the VLP entry efficiency was significantly decreased to 5% at 70 μM, the highest working concentration of CPZ (Figure 2C). That is to say, 95, 80, and 70% VLP were inhibited to enter into SB cells by 70, 7, and 1 μM CPZ, respectively. The effect of CPZ on VLP entry was dose-dependent.

Dynamin is a cellular GTPase which is essential for CCV formation and is required for membrane budding at a late stage during the transition from a fully formed pit to a pinched-off vesicle. Dynasore acts as a potent and specific inhibitor of dynamin by rapidly blocking coated vesicle formation within seconds of dynasore addition [51]. We therefore used different concentrations of dynasore (1, 10, and 80 μM) to treat SB cells to validate the role of dynamin in VLP entry. The fluorescent signals were significantly reduced in dynasore-treated cells (Figure 2B) and the VLP entry efficiency was greatly reduced in the presence of dynasore (Figure 2D) in a dose-dependent manner (71.4, 48.5, and 40%).

Since we obtained the preliminary result that CME is involved in RBS entry, cell perturbation assays using siRNA silencing were performed to knockdown the expression of the SB cell clathrin heavy chain (sbCH) and light chain-A (sbCL) to evaluate the dependence of clathrin for VLP entry. The cDNA sequences of sbCH and sbCL were cloned and sequenced (accession Num: KX989459, KX989460). BLAST homology and SMART analysis showed that the deduced amino acid sequences of sbCH and sbCL matched well with the clathrin from other species. Their predicted domains also shared similar architectures with other species as shown in Additional file 1. The siRNA specific to sbCH (siCH) and sbCL (siCL) were designed (Table 2) and synthesized. The siRNA for non-inhibition control with an irrelevant sequence (siIR) to any available data were also included. The siRNA were first transfected into SB cells for testing silence efficiency. siCH and siCL decreased the expression of sbCH and sbCL more than 50%, respectively (data not shown). After 48 h post-transfection, VLP entry assays were performed on transfected SB cells that were either fixed for IFA or collected for ELISA. As shown in Figure 2E, the fluorescent signal of VLP in siCH and siCL transfected cells were reduced, whereas that in siIR transfected cells was similar to the untreated control. The silencing of sbCH and sbCL also greatly reduced the VLP entry efficiency to 28.5 and 42.8% respectively compared with the siIR control (Figure 2F). Taken together, these results establish that dynamin-dependent and CME are involved in RBS entry into SB cells.

### RBS entry is dependent on fluidity of cholesterol but is not involved with caveolae/raft-dependent endocytosis

**Figure 3:**
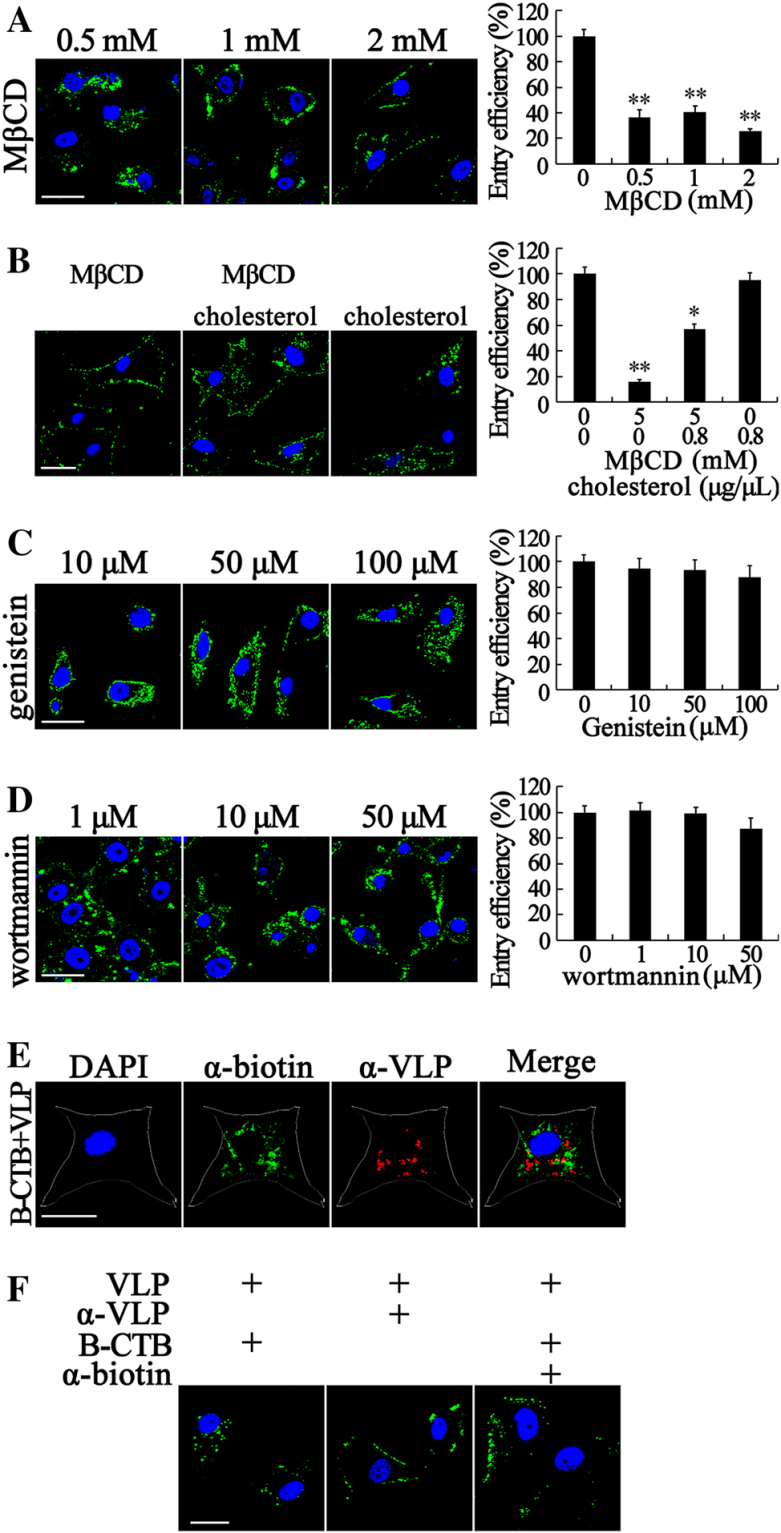
**VLP entry is dependent on fluidity of cholesterol but is not involved with caveolae/raft-dependent endocytosis. A** Depletion of cholesterol from the plasma membrane inhibited RBS entry. SB cells were pretreated with MβCD at the indicated doses for 1 h prior to incubation with RBS. VLP entry assays, IFA, fluorescent microscopy and ELISA were performed. **B** Cholesterol replenishment partially recovered CGV entry. High concentration (5 mM) MβCD-treated cells were incubated without or with exogenous cholesterol for 1 h followed by CGV entry assay. After fixation, fluorescent microscopy was performed directly to determine the CGV location. ELISA was also performed to show the entry efficiencies in different treatments. **C**, **D** Block of the tyrosine kinase signal by genistein and PI3/4 K activity by wortmannin did not inhibit RBS entry. Inhibitors of genistein (**C**) and wortmannin (**D**) at indicated concentrations were used to treat SB cells followed by RBS entry. For **A**, **C**, and **D**, RBS (green) in cells were visualized by IFA and cell nuclei (blue) were stained by DAPI. The entry efficiency was examined by ELISA and the data shown are the mean ± SD of the results from three independent experiments. **E** RBS entry is dependent on cholesterol but not on sphingolipids. B-CTB and RBS were simultaneously incubated with SB cells to perform entry assay for 4 h and their subcellular localization were determined by IFA using anti-biotin (α-biotin, green) and anti-VLP (α-VLP, red) as primary antibody. The outline of the cell was indicated by the white line. The merge panel displayed that most of B-CTB and VLP were localized in different subcellular locations. **F** CGV entry is dependent on fluidity of cholesterol. CGV entry assays were performed for 2 h with different additives as indicated above the panels. After being washed and fixed, cells were observed by confocal microscopy. The bars indicate 25 μm. *, *p* < 0.05, **, *p* < 0.01.

Cholesterol is very important to the caveolae/raft-dependent endocytosis. Thus, the chemical agents filipin, nystatin, or methyl-b-cyclodextrin (MβCD) that can sequester or deplete cholesterol from the plasma membrane are usually used to inhibit the virus entry into cells through caveolae/raft-dependent endocytosis [1]. However, cholesterol is also an essential factor to CME. Cholesterol can regulate the fluidity of plasma membrane, which can affect formation of CCV. We therefore pretreated the SB cells with different concentrations of MβCD before VLP entry assay to investigate whether RBS entry is dependent on cholesterol. As expected, 2, 1, and 0.5 mM MβCD inhibited 74.4, 59.5, and 63.6% RBS entry into SB cells, respectively (Figure 3A). We found that most parts of RBS stayed outside of plasma membranes in high concentration MβCD-treated cells. By contrast, a small amount of RBS reached the perinuclear area (Figure 3A, 2 mM) compared with no treatment control.

To confirm that the inhibitory effects for RBS entry were caused by cholesterol depletion, cell membrane cholesterol was replenished with high concentration of exogenous cholesterol, and the recovery of RBS entry was analyzed. In this replenishing experiment, CGV was used to perform VLP entry and was directly visualized by fluorescent microscopy after cell fixation. Therefore, the fluorescent signal of CGV is relatively weaker than that of RBS in IFA. As shown in Figure 3B, almost all of the CGV particles were settled on the plasma membrane of MβCD-treated cells at the high concentration (5 mM). Cholesterol-depleted cells (pretreated with 5 mM MβCD) were incubated with exogenous cholesterol followed by VLP entry assay. The CGV were found in cells and on the plasma membrane (Figure 3B). The inhibitory effect was partially reversed with cholesterol replenishment at a concentration of 0.8 μg/μL. The entry efficiency of cholesterol replenishment samples was restored to a mean of 57% compared with the mock-treated cells. Interestingly, cholesterol depletion resulted in a reduction but not abolishment of CGV entry. By contrast, the supplement of cholesterol in no MβCD-treated cells did not enhance the entry of CGV. CGV entry may also occur at low cholesterol levels, but increased cholesterol shows no difference of this process.

Endocytosis via caveolae is dynamin dependent, sensitive to cholesterol depletion, and associated with signaling events. In this case RBS entry is dependent on dynamin and cholesterol. Thus, whether the entry involved caveolae/raft-dependent endocytosis should be identified. The effects of genistein and wortmannin on VLP entry were determined as caveolae budding is regulated by reversible phosphorylation [52]. Previous research on ISKNV [17] and tiger frog virus (TFV) [53] showed that signal induction is important for viral entry. Genistein, a tyrosine kinase inhibitor, blocks the signals induced by ISKNV and TFV. Thus, we determined whether this chemical was also capable of blocking the entry of RBS. As shown in Figure 3C, the genistein treatment did not block VLP entry even at a high concentration (100 μM). The entry quantity of VLP (88%) exhibited no significant difference when compared with mock-treated control. This result indicates that the transmembrane signaling is not important for RBS entry into SB cells.

The inhibitory regulative G protein (Gi)-coupled receptor was found abundantly in caveolae. Moreover, the activation of the Gi-coupled Src kinase pathway plays an important role in the formation and migration of endocytosis vesicles in the caveolae/rafe-dependent pathway [54]. Wortmannin was used as an inhibitor specific to phosphoinositide 3/4-kinase (PI3/4K) leading to reduction of PI3P content. PI3K/Akt Signal pathway is very closely correlated with the caveolae/raft-dependent endocytic pathway. PI3K is also a phospholipid kinase, such as PI(3,4)P, PI(4,5)P2. Some studies report that these phospholipids are regulators of CME [1]. Thus, SB cells were treated with different concentrations of wortmannin and VLP entry assays were performed. As shown in Figure 3D, 50, 10, and 1 μM wortmannin could block 23.0, 0.82, and −1.4% RBS entering into SB cells compared with the mock-treated control. Inhibition of PI3/4K activity exhibits no perceptible effect on preventing RBS entry.

Lipid rafts are subdomains of the plasma membrane that contain high concentrations of cholesterol and sphingolipids. CTB binds to the sphingolipid GM1, which is a marker to identify lipid rafts [55]. To further illustrate the endocytosis of RBS, clathrin-independent and cholesterol-sensitive CTB [52] was used as the control for subcellular localization via caveolae/raft-dependent endocytosis. Biotin conjugated CTB (B-CTB) and RBS were simultaneously incubated with SB cells to perform the entry assay for 4 h. Their subcellular localizations were determined by IFA using anti-biotin and anti-VLP antibodies as primary antibody. As shown in Figure 3E, both B-CTB (green) and RBS (red) stayed at the perinuclear area. However, a few of them were co-localized as indicated in the merged panel. This result revealed that both B-CTB and RBS entries required lipid rafts, in which B-CTB needed sphingolipids and VLP depended on cholesterol, and they used different endocytosis pathways. Therefore, RBS entry is not involved with caveolae/raft-dependent endocytosis.

Based on the above results, we found that RBS entry is clathrin-dependent but caveolin-independent, cholesterol-depletion sensitive but insusceptible to replenishment, and not co-localized with sphingolipids. Accordingly, we presumed that VLP entry may be mediated by the protein receptor(s) located at lipid rafts or even use specific lipids as the receptor. The fluidity of cholesterol and sphingolipids is important for VLP entry because the binding of the receptors and their ligands (virus or toxin) and their internalization are sensitive to the fluidity of the plasma membrane [56]. Components of lipid rafts maintain certain mobility on the plasma membrane. For example, individual gangliosides diffuse in the plasma membrane of cells at a diffusion constant in the range of a few mm^2^/s, interaction with cholesterol leads to short-lived confinement in the nanometer range [57], and the binding of CTx to GM1 strongly reduces the diffusion of other lipid molecules in the same membrane as well as increases the melting temperature of supported membrane bilayers [28]. Thus, we used B-CTB to bind the raft-resident ganglioside GM1 and anti-biotin antibodies were used to bind B-CTB, mediating blockage of B-CTB entry and reduction of lipid raft fluidity. At the same time, entry assays using CGV were performed to evaluate the influence to CGV movement at low fluidity of plasma membrane lipids. After 2 h of CGV entry assays with different additives as indicated in Figure 3F, cells were washed extensively, fixed, and checked by confocal microscopy. As expected, most of the CGV stayed at the plasma membrane with little entry when B-CTB and anti-biotin antibody were added together (Figure 3F, right panel). By contrast, the cells without antibody and with CGV plus B-CTB (Figure 3F, left panel) showed the normal entry property of CGV. The cells with CGV and anti-VLP antibody (Figure 3F, middle panel) exhibited the membrane settlement of the blocked CGV. In summary, RBS entry is dependent on fluidity of cholesterol but is not involved with caveolae/raft-dependent endocytosis. However, the entry receptor(s) for OGNNV is worthy of further study.

### Entry of RBS is independent of macropinocytosis

**Figure 4:**
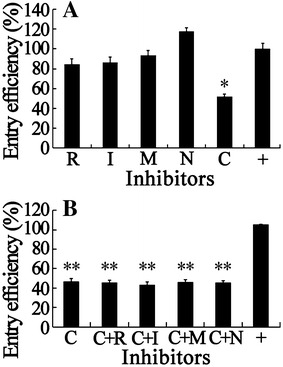
**Entry of RBS is independent of macropinocytosis. A** Inhibitors of macropinocytosis were used individually. **B** Inhibitors of macropinocytosis and CME were used simultaneously. All inhibitors of macropinocytosis (40 μM) including Rottlerin (R), IPA-3 (I), ML-7 (M), and NSC23766 (N), and CME inhibitor (7 μM) CPZ (C) were used, alone or overlay as indicated +, to treat SB cells for 1 h. Two hours of VLP entry assays with the inhibitors and the following ELISA were performed. RBS entry without inhibitor was served as the untreated control (+). *, *p* < 0.05, **, *p* < 0.01.

Besides the clathrin- and caveolae/raft-dependent endocytic processes, macropinocytosis has received increasing attention because of its roles in immune defense and virus entry [2]. Increasing numbers of studies have reported that macropinocytosis requires signaling events, especially in the uptake of virus particles. During influenza virus entry, a functional actomyosin network and the activation of p21-activated kinase 1 (PAK1) are essential for macropinocytosis, while EV1 and vaccinia virus entry via macropinocytosis depend on PAK1 and protein kinase C (PKC) [58, 59]. A previous report demonstrated that macropinocytosis is an important route for the cellular entry of DGNNV [41]. Therefore, in our study, ML-7 was used to antagonize the phosphorylation of myosin light chain kinase to regulate myosin II activity, and NSC23766 was used to specifically inhibit Rac1 activity without affecting the closely related Rho-GTPases. We also used rottlerin and IPA-3 to inhibit PKC and PAK1 activities in SB cells, respectively. As shown in Figure 4A, rottlerin (R), IPA-3 (I), ML-7 (M), and NSC23766 (N), all exhibited no statistically significant change on the percentage of RBS entry efficiency whereas CPZ (C), the inhibitor for CME, dramatically reduced the entry efficiency compared with that of untreated cells (+). The locations of entered RBS in these four inhibitor treated cells were similar to that in untreated cells (data not shown). These results suggest that multiple signaling molecules were not involved in RBS entry, which agree with the results of kinase inhibitors, genistein and wortmannin.

Although the inhibitors of macropinocytosis do not block RBS entry with significant difference compared with the untreated cells, a certain reduction can be seen in samples treated with rottlerin, IPA-3, and ML-7. The compensatory activation of alternative pathways may appear when one pathway is closed down [1]. Therefore, we tried to determine whether a statistical difference exists when inhibitors of macropinocytosis and CME are used simultaneously. As shown in Figure 4B, no obvious difference was observed between the entry efficiency in CPZ-treated cells and the CPZ plus macropinocytosis inhibitor-treated cells. In summary, these results indicate that entry of RBS is independent of macropinocytosis.

### RBS entry is pH dependent

**Figure 5:**
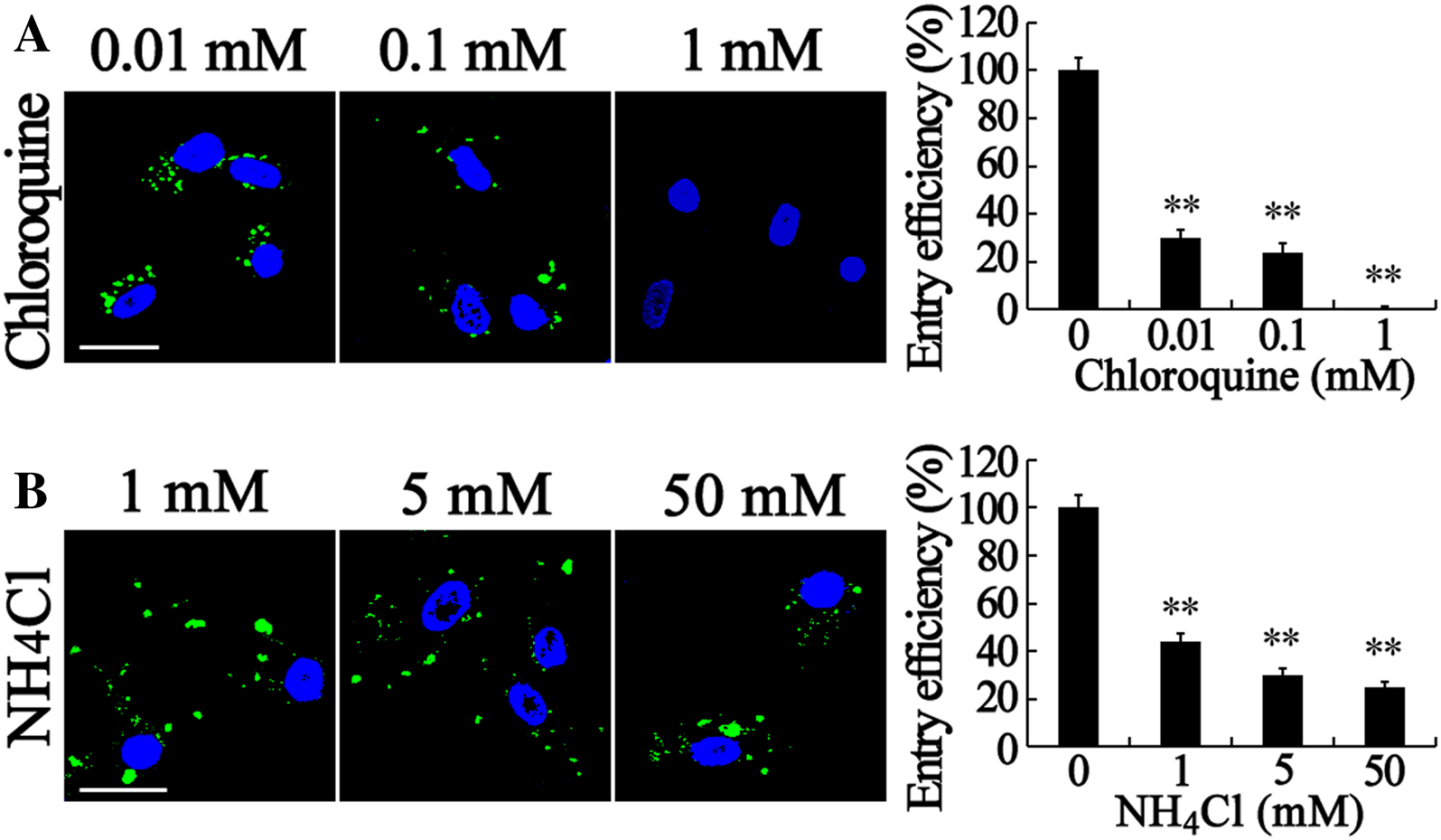
**Inhibition of endosomal acidification prevents RBS entry.** SB cells were treated with chloroquine (**A**) or NH_4_Cl (**B**) at the indicated doses for 1 h. VLP entry assays, IFA, fluorescent microscopy, and ELISA were performed. RBS (green) in cells were visualized by IFA and cell nuclei (blue) were stained by DAPI. The entry efficiency was examined by ELISA and the data shown are the mean ± SD of the results from three independent experiments. The bars indicate 25 μm. **, *p* < 0.01.

Once internalized within the primary endocytic vesicles, many viruses follow the intracellular pathways of the endosomal system which is responsible for molecular sorting, recycling, degradation, storage, and processing [1]. In the endosomal system, a gradual drop in internal pH occurs from mild acidity (pH 6.5–6.0) in early endosomes to values below 5 in endolysosomes. Virus entry via CME is sensitive to pH changes, indicating the importance of low pH for viral infection [60]. To further analyze the role of low pH in RBS entry, lysosomotropic agents, such as chloroquine and NH_4_Cl, the chemical inhibitors for endosomal acidification, were used to treat SB cells. As shown in Figure 5, different concentrations of chloroquine and NH_4_Cl significantly decreased the VLP entry efficiency compared with no treated cells. The high concentration of chloroquine (1 mM) can totally block the entry (Figure 5A). Interestingly, most of the entered RBS in treated cells were stuck in the vesicle-like spots that were larger than the small dots in untreated cells (Figure 2A). VLP quantitation revealed that 0.01 mM chloroquine can block more than 70% RBS. In addition, 1 mM chloroquine can totally block VLP entry, conforming to the IFA results. Up to 50 mM NH_4_Cl can reduce the VLP entry efficiency to 25% (Figure 5B). These results demonstrate that RBS enter SB cells in a pH-dependent manner.

### RBS entry is dependent on the cytoskeleton

**Figure 6:**
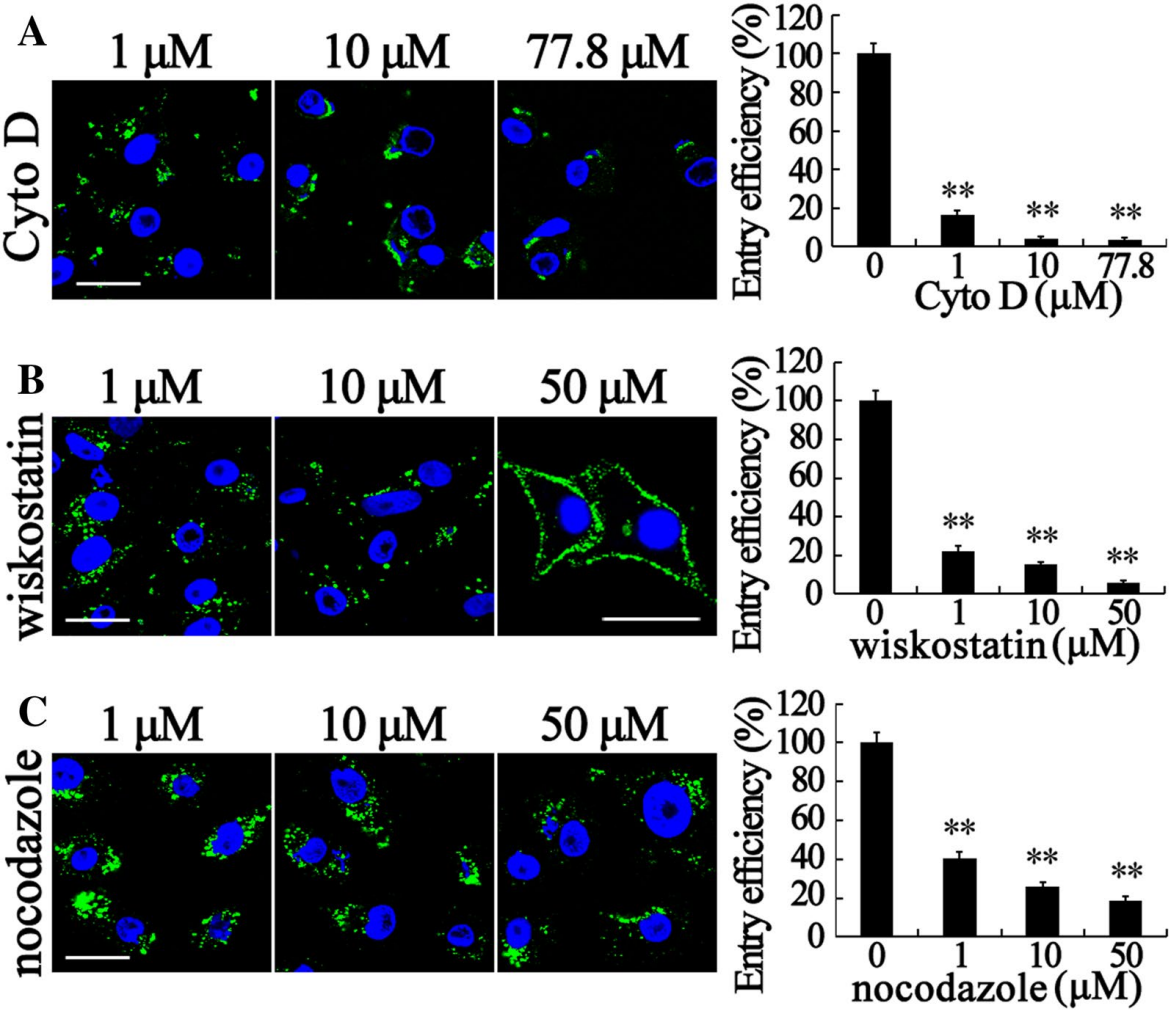
**RBS entry into SB cells is dependent on cytoskeleton.** SB cells were treated with chemical inhibitors of CytoD (**A**), wiskostatin (**B**), and nocodazole (**C**) respectively at the indicated doses for 1 h. VLP entry assays, IFA, fluorescent microscopy, and ELISA were performed. RBS (green) in cells were visualized by IFA and cell nuclei (blue) were stained by DAPI. The entry efficiency was examined by ELISA and the data shown are the mean ± SD of the results from three independent experiments. The bars indicate 25 μm. **, *p* < 0.01.

Upon internalization, viruses are usually delivered to endosomal compartments and transported to the replication sites along actin filaments/microtubules [61, 62]. To determine the roles of actin filaments/microtubules during RBS entry, several chemical inhibitors were used as follows. CytD inhibits actin filament elongation at the barbed end and wiskostatin blocks neuronal Wiskott-Aldrich syndrome protein (N-WASP) activity by stabilizing its autoinhibited conformation, thus leading to a significant decrease in the rate of actin polymerization [63]. Nocodazole depolymerizes microtubules by preventing the formation of one of the two interchain disulfide linkages. As shown in Figure 6, disruption of actin dynamics by CytD and wiskostatin significantly reduced the total number of RBS entered in SB cells. Up to 77.8, 10, and 1 μM CytD can block 96.5, 96.0, and 83.2% RBS entry into SB cells, respectively (Figure 6A). By contrast, 50, 10, and 1 μM wiskostatin can block 94.2, 84.6, and 77.9% RBS entry into SB cells, respectively (Figure 6B). It is worthwhile to note that most VLP stayed around the plasma membrane in 50 μM wiskostatin treated cells although the inhibition of entry efficiency was lower in wiskostatin treated samples than in CytD treated samples. Furthermore, we treated SB cells with nocodazole. The results revealed that 50, 5, and 1 μM nocodazole can block 81.3, 74, and 59.4% RBS entry into SB cells (Figure 6C). These results suggest that RBS entry into SB cells is dependent on cytoskeleton of actin filaments and microtubules.

### RBS enter NNV-sensitive cells via CME

**Figure 7:**
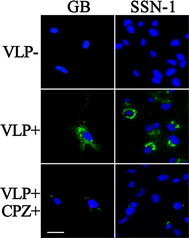
**RBS enters GB and SSN-1 cells via CME.** VLP entry assay, IFA and fluorescent microscopy were performed on ten cell lines and RBS can only enter GB and SSN-1 as indicated in VLP + panels. To identify the endocytic pathway, GB and SSN-1 cells were pretreated with CPZ for 1 h. VLP entry assay, IFA and fluorescent microscopy were performed and RBS entry was dramatically decreased in VLP +/CPZ + samples. RBS (green) in cells were visualized by IFA and cell nuclei (blue) were stained by DAPI. The bars indicate 25 μm.

Virus-like particles are robust protein cages that mimic the overall structure of the native virions but lack the viral genome. They are often antigenically indistinguishable from the native virus but the entry performance might be slightly different with native virus [64]. To determine the entry property of RBS, VLP entry assay and IFA were used to screen several types of cell lines including fish, mammals, and insects. A total of 10 cell lines were used for screening, namely, SSN-1, GB, FHM, EPC, MFF, Hela, 293T, BHK, Sf9, and S2. The entry assay and IFA revealed that RBS only entered SSN-1 and GB cells (Figure 7) that are sensitive to NNV infection (personal communications) but did not enter other tested cell lines even the fish cell lines (data not shown). The results suggest that the entry property of RBS is similar to that of native NNV.

Since we confirmed that CME is involved in RBS entry into SB cells as well as RBS only entering SSN-1 and GB, we determined the VLP endocytosis pathway in SSN-1 and GB. During the entry assay, we found that RBS entered into SSN-1 and GB cells quickly and reached the perinuclear regions within 90 min (Figure 7). Furthermore, CPZ was used to analyze the role of CME during RBS entry into SSN-1 and GB cells. As shown in Figure 7, VLP entry efficiencies in CPZ treated samples dramatically decreased when compared with untreated samples. This result suggests that RBS entered SSN-1 and GB cells via CME. In other words, RBS entry into NNV-sensitive cells is dependent on CME.

## Discussion

Viruses use endocytosis to effectively enter host cells and then transport to suitable endosomes or other intracellular organelles for the subsequent genome release or replication. Studying virus entry pathways is important to understand viral pathogenesis and to develop effective drugs to block virus infection at the very early and critical step, that is, virus entry [1].

In research on virus entry, besides native virus, recombinant or modified viruses and VLP are also used [33, 41, 65]. VLP can be produced eukaryotically and prokaryotically, and can also be easily modified by genetic engineering. Therefore, the high stability of genetics and production can be confirmed, avoiding the low-mutation rate occurring in wild-type virus [44]. Furthermore, the advantages of easy and large production of VLP are beneficial to perform parallel experiments using multiple chemical inhibitors at the same time. As a result, credible data on the situation of equal status of culturing cells can be obtained. Although detecting the expression of the viral gene at a certain period after native virus infection in the entry perturbation assay is a highly sensitive method, it is difficult to distinguish whether effective blocking is due to entry inhibition or because of late-stage virus replication inhibition [1]. In this study, we replaced native virus with RBS and CGV (modified VLP) to perform the entry assay. VLP can show the entry status on quantity and location as well as avoid the long time wait when detecting viral gene expression after infection. Nevertheless, between RBS/CGV and authentic NNV, maybe there are some slight differences which might affect the sophisticated research on virus entry and entry receptor. In the study of RBS, we proved that the entry ability of RBS/CGV is the same as native NNV. Therefore, the RBS-SB cell entry model is reliable and stable.

Macropinocytosis is one of the pathways during virus entry of DGNNV [41]. In the RBS-SB model, we proved that macropinocytosis is not involved in RBS entry into SB cells. This result is proven by biochemical perturbation assays regardless of using macropinocytosis inhibitors only or overlay using the CME inhibitor. We also screened 10 more cell lines from different species by VLP entry assays. We demonstrated that RBS can only enter NNV-sensitive cells and mainly by CME. Moreover, in another NNV receptor relative study, the earliest detection time point for gene expression was set to 1 h post-infection [40], indicating that NNV entry is a quick process in accordance with the characteristic of CME. We also believed that a minor portion of RBS enters SB cells via macropinocytosis but that portion is predicted below 1% of all entered virus. This result was consistent with the fact that RBS is soaked randomly by the normal cell macropinocytosis.

The structure and composition of virus are simple but their interactions with host cells are complex, especially the entry process. Besides endocytic pathways, some cell components or conditions are essential for successful virus entry [1]. In our study, the entry of RBS into SB cells was significantly and dose-dependently reduced by treatment with CPZ and dynasore as the key inhibitors for CME. Furthermore, the VLP entry efficiency was also dramatically decreased by silencing the SB clathrin heavy and light chains. We basically confirmed that RBS enter SB cells mainly via CME depending on dynamin-2.

Lipid rafts are involved in many cellular processes, such as endocytosis, signaling, protein sorting, and intracellular membrane trafficking. Sphingolipids are a major class of membrane lipids and are also involved in the stability of the lipid scaffold of lipid enriched microdomains as a bulk lipid. Cholesterol is a ubiquitous component of eukaryotic cell plasma membrane and is central to the organization, dynamics, function, and sorting of lipid bilayers in vivo [66]. In our study, cholesterol is not only essential for RBS/CGV entry as demonstrated in assays of cholesterol depletion and replenishment but also its fluidity is necessary for effective RBS entry as shown in B-CTB binding assay. In the depletion and replenishment assays, we can observe that the depletion of cholesterol cannot abolish RBS entry and the replenishment of cholesterol can only partially recover the depletion of MβCD. The excess cholesterol cannot enhance CGV entry, suggesting that the quantity of cholesterol is not the most important factor during VLP entry. Therefore, we suspected that the fluidity of cholesterol is the key element. B-CTB and anti-biotin antibody were spontaneously used to decrease the fluidity of sphingolipid, eventually reducing the cell membrane fluidity [28, 56]. The results show that RBS entry was blocked and mainly stayed at plasma membrane when anti-biotin antibody was present, demonstrating that the low fluidity of lipid raft greatly reduced RBS entry. Accordingly, the fluidity of cholesterol on plasma membrane is crucial for RBS entry. Furthermore, the cellular localizations of RBS and B-CTB were largely not the same, suggesting the different endocytic pathways and intracellular trafficking routes of RBS and CTB. The result also verified that caveolae/raft-dependent pathways mediate the internalization of sphingolipids and sphingolipid-binding toxins, such as CTB as indicated in previous studies [52]. Actually, RBS entry requires cholesterol and membrane fluidity is closely related to the cellular receptor(s)-mediated virus entry. Previous studies showed that the functional cellular components including HSP70 [40] and extracellular sialic acid [41] are required for active NNV infection. In addition, we performed the experiment that extracellular components of SB cells were destroyed by several reagents before the VLP entry assay. The results show that only the treatments of protease K and neuraminidase can block RBS entry (unpublished data). It can be deduced that the most important component on the cell surface mediating virus entry is protein with sialic acid modification. Therefore, the cellular entry receptor for NNV is basically glycoprotein. It is worthy of further study to determine whether the low fluidity of lipid raft restricts the mobility of entry receptor(s), mediating the blockage of NNV entry. Nevertheless, we also cannot rule out the possibility that other components in lipid rafts are the authentic entry receptor(s) for NNV entry.

Once internalized within primary endocytic vesicles, the incoming viruses will follow the intracellular pathways, the endosomal system, to the replication sites. The pH-dependent endosomal system is responsible for molecular sorting, recycling, degradation, storage, processing, and transcytosis of incoming substances [1]. Therefore, we studied whether RBS entry is dependent on low pH after the endocytosis pathway since RBS was confirmed to be CME. Our results show that chloroquine and NH_4_Cl, the lysosomotropic weak bases that decrease the pH of the endosomal system, can significantly block RBS entry, suggesting that RBS is delivered to the perinuclear area through the endosomal system. The intensive studies on the interaction of VLP and endosome network and the intracellular trafficking pathway should be further investigated. For example, the co-localization study of RBS and the markers of the main organelles of the endosome network, such as Rab5 (early endosome), Rab7 (late endosome), Rab22 (recycling endosome), and Rab9 (trans-Golgi network) should be performed [1]. The migration of endosomes from peripheral area to the perinuclear area and finally fusion with lysosomes is mediated by the change in the interaction with cytoskeletal elements. Thus, the normal function of cytoskeleton is fundamental for active virus infection via endocytosis. To verify whether RBS entry is dependent on cytoskeleton, we used CytD and wiskostatin to disrupt actin dynamics and nocodazole to depolymerize microtubules. All these three reagents dramatically blocked RBS entry, suggesting that both actin filaments and microtubules are important for RBS entry. Interestingly, high concentration of wiskostatin (50 μM) inhibited RBS entry but did not affect the RBS binding to cells as shown by the plasma membrane surface localization of RBS. According to previous studies [38] and our unpublished data, the genome replication of NNV occurred at the virus factory in the mitochondria. Therefore, the intracellular trafficking of NNV in sensitive cells remains to be fully elucidated.

Betanodaviruses have strong infectivity to a wide range of hosts [38, 44]. In this study, we determined the entry property of RBS by screening 10 cell lines via VLP entry assay and IFA. RBS only entered SSN-1 and GB cells that are sensitive to NNV infection but did not enter other tested cell lines. This result suggests that the entry property of VLP is the same as that of native NNV. For Hela and 293T cells, we did not observe the VLP entry or attachment in the assays, which was different from the results of a previous study showing that red-spotted grouper nervous necrosis virus can attach but cannot penetrate the cells [67]. Furthermore, the CPZ pretreatment of SSN-1 and GB cells showed that RBS entry significantly decreased, indicating a common feature that RBS entry into NNV-sensitive cells is dependent on CME.

In conclusion, the evidence presented here demonstrates that RBS enters SB cells via CME, depending on dynamin-2, cholesterol and its fluidity, low pH, and cytoskeleton. As a virus in the same family and different genus, the entry pathway studies of betanodavirus are more intensive than that of flock house virus (FHV), the representative species of alphanodavirus, because the entry studies of FHV focused on the structure transformation of virion during virus-cell interaction and the dependency of the acidic endocytic pathway [39]. However, the specific endocytic pathway and entry receptor(s) for FHV are yet to be clearly defined. Aquatic viruses use various entry pathways depending on the virus, such as frog virus 3 (FV3) [68], SGIV [2] and infectious hematopoietic necrosis virus [69] utilizing CME, TFV [53] and ISKNV [17] employing caveolae/raft-dependent endocytosis, indicating the complicated virus-cell interaction. The different endocytic pathways used by TFV, FV3 and SGIV that are closely related viruses in the same genus may be due to different cell lines utilized in entry studies, simultaneously suggesting the broad host range of ranavirus. Nevertheless, in our study, the fact that RBS enters both three NNV-sensitive cell lines via CME depending on cholesterol indicates the unity of the entry pathway in betanodavirus. Further studies are needed to determine the detailed mechanisms involved in the entry routes for betanodavirus and the specific receptor(s) mediating betanodavirus entry.

## Additional file

**Additional file 1.** **The predicted protein domains of sbCH and sbCL by SMART.** CLH means clathrin heavy chain while Clathrin_lg_ch indicates clathrin. The pink block indicates the low complexity. The digits show the length of the linear proteins.

## Abbreviations

OGNNV: orange-spotted grouper nervous necrosis virus
VLP: virus-like particle
RBS: OGNNV VLP
CGV: C terminal GFP-tagged virus-like particle
GFP: green fluorescent protein
SB cell: Asian sea bass fibroblast cell
GB cells: grouper brain cells
SSN-1 cell: striped snakehead cell
FHM cell: fathead minnow cell
EPC cell: *epithelioma papulosum cyprini* cell
MFF-1 cell: mandarin fish fry cell
BHK cell: baby hamster kidney cell
BSA: bovine serum albumin
IFA: indirect immunofluorescence assays
FHV: flock house virus
SGIV: Singapore grouper iridovirus
SV40: simian virus 40
EV1: echovirus 1
ISKNV: infectious spleen and kidney necrosis virus
JEV: Japanese encephalitis virus
HCV: hepatitis C virus
DGNNV: dragon grouper nervous necrosis virus
TFV: tiger frog virus
CME: clathrin-mediated endocytosis
CCP: clathrin-coated pit
CCV: clathrin-coated vesicle
CPZ: chlorpromazine
MβCD: methyl-b-cyclodextrin
CTx: cholera toxin
CTB: cholera toxin B subunit
CytD: cytochalasin D
CHC: clathrin heavy chain
CLC: clathrin light chain
