# Supplementary figures and images for: Betanodavirus-like particles enter host cells via clathrin-mediated endocytosis in a cholesterol-, pH- and cytoskeleton-dependent manner
Source: Vet Res. 2017 Feb 8;48:8. doi: 10.1186/s13567-017-0412-y (PMC5299686; doi:10.1186/s13567-017-0412-y)

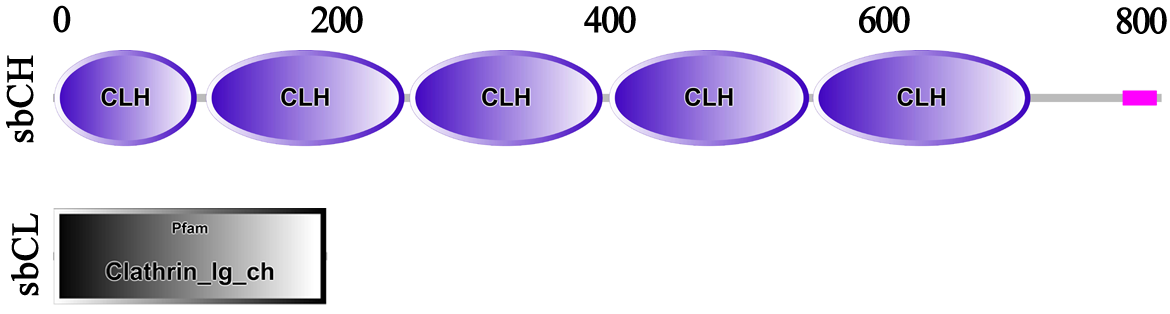

Supplement: Supplementary file 1 — Additional file 1. The predicted protein domains of sbCH and sbCL by SMART. CLH means clathrin heavy chain while Clathrin_lg_ch indicates clathrin. The pink block indicates the low complexity. The digits show the length of the linear proteins. [file 13567_2017_412_MOESM1_ESM.tif]
